# Circular and Leaderless Bacteriocins: Biosynthesis, Mode of Action, Applications, and Prospects

**DOI:** 10.3389/fmicb.2018.02085

**Published:** 2018-09-04

**Authors:** Rodney H. Perez, Takeshi Zendo, Kenji Sonomoto

**Affiliations:** ^1^Department of Bioscience and Biotechnology, Faculty of Agriculture, Graduate School, Kyushu University, Fukuoka, Japan; ^2^National Institute of Molecular Biology and Biotechnology, University of the Philippines Los Baños, Los Baños, Philippines

**Keywords:** lactic acid bacteria, bacteriocins, circular bacteriocins, leaderless bacteriocins, bacteriocin biosynthesis, mode of action

## Abstract

Bacteriocins are a huge family of ribosomally synthesized peptides known to exhibit a range of bioactivities, most predominantly antibacterial activities. Bacteriocins from lactic acid bacteria are of particular interest due to the latter’s association to food fermentation and the general notion of them to be safe. Among the family of bacteriocins, the groups known as circular bacteriocins and leaderless bacteriocins are gaining more attention due to their enormous potential for industrial application. Circular bacteriocins and leaderless bacteriocins, arguably the least understood groups of bacteriocins, possess distinctively peculiar characteristics in their structures and biosynthetic mechanisms. Circular bacteriocins have N-to-C- terminal covalent linkage forming a structurally distinct circular peptide backbone. The circular nature of their structures provides them superior stability against various stresses compared to most linear bacteriocins. The molecular mechanism of their biosynthesis, albeit has remained poorly understood, is believed to possesses huge application prospect as it can serve as scaffold in bioengineering other biologically important peptides. On the other hand, while most bacteriocins are synthesized as inactive precursor peptides, which possess an N-terminal leader peptide attached to a C-terminal propeptide, leaderless bacteriocins are atypical as they do not have an N-terminal leader peptide, hence the name. Leaderless bacteriocins are active right after translation as they do not undergo any post-translational processing common to other groups of bacteriocins. This “simplicity” in the biosynthesis of leaderless bacteriocins offers a huge commercial potential as scale-up production systems are considerably easier to assemble. In this review, we summarize the current studies of both circular and leaderless bacteriocins, highlighting the progress in understanding their biosynthesis, mode of action, application and their prospects.

## Introduction

Over the past decade, there has been a growing interest on bioactive peptides of ribosomal origin also known as ribosomally synthesized and post-translationally modified peptides (RiPPs) produced by a wide range of bacterial species (Arnison et al., 2013). Although, many bacterial species also synthesize bioactive compounds non-ribosomally, more attention is given to RiPPs as they offer clear advantages such as the relative ease in establishing of their future scale-up production systems as well as their high amenability to bioengineering (Perez et al., 2014). Perhaps one of the most popular members of the diverse group of RiPPs are the family bacteriocins. Bacteriocins are estimated to be produced by more than half to almost all bacterial species (Riley and Wertz, 2002). However, bacteriocins produced by lactic acid bacteria (LAB) are of more particular interest due to the latter’s association to food fermentation and their general notion to be safe (Perez et al., 2014). Bacteriocins are ribosomally synthesized by their respective producer strains in order to gain advantage in their competitive ecological niche. Although most bacteriocins were isolated and studied for their antimicrobial activities these compounds were also found to exhibit diverse remarkable bioactivities, thus creating new research fields and expanding the areas of their potential applications. For a more detailed review on the various bioactivities of bacteriocins, one should refer to the recent commentary by Drider et al. (2016).

In the era of genomics, wherein bacteriocin-dedicated databases and online platforms with diverse tool for the automated characterization (Hammami et al., 2010) and screening of bacteriocin gene clusters (Medema et al., 2011; van Heel et al., 2013) are now available, the discovery of new novel bacteriocins is expected to accelerate further. The continuing interest and the rapidly growing repertoire of bacteriocins have led to some confusion on their classification schemes. Since the first classification proposed by Klaenhammer (1993), many other classification schemes have been suggested. The most recent scheme by Alvarez-Sieiro et al. (2016) is a further modification of the previous widely accepted classification scheme by Cotter et al. (2005). However, in this review, we will not attempt to resolve the differences in these various classification schemes so as not to further muddle this mooted issue. In any case, it is apparent that in all proposed classification schemes two distinct groups, the circular bacteriocins and leaderless bacteriocins, are clustered in their respective sub-classes on the basis of their unique characteristics. These two groups of bacteriocins have so far remained the most poorly understood, yet arguably the most promising group of bacteriocins.

Circular bacteriocins are a family of RiPPs that are clustered based on their structurally conserved circular peptide backbone arising from the covalent linkage of their N- and C-terminal residues. In this review, the term “circular” (and circularization for the process of) should not be confused with the cyclic structures of non-C- and N-terminal ligated bioactive peptides such as that of other bacteriocins containing lanthionine rings and disulfide bridges. This should also be clearly distinguished from the cyclic structures found in many non-ribosomally antimicrobial peptides. The most remarkable property of circular bacteriocins is their superior stability compared to their linear counterparts. But perhaps the greatest promise of this group of bacteriocins lies on the understanding of their biosynthetic mechanism, although still has remained the biggest challenge.

On the other hand, leaderless bacteriocins are peculiarly different from other bacteriocins. While most bacteriocins are synthesized as an inactive precursor peptide containing N-terminal leader moiety, leaderless bacteriocins are synthesized without these leader sequences hence the term. This peculiar characteristic of leaderless bacteriocins disqualifies them in the family of RiPPs as they do not undergo any post-translational modifications. Nonetheless, the ribosomal nature of their biosynthesis and their innate “bacteriocin” characteristics makes them undeniably a close relative to most RiPPs. Moreover, the simplicity of leaderless bacteriocins holds their most remarkable potential as it makes them easily adaptable to any expression systems for their eventual scale-up production systems.

This review summarizes these two emerging groups of bacteriocins highlighting the progress in our understanding of their biosynthesis, mode of action, application and their prospects.

## Circular Bacteriocins

Head-to-tail ligated RiPPs are not limited to bacterial origin as many circular antimicrobial compounds have been found from many eukaryotic organisms such as mammals, plants, and fungi (Conlan et al., 2010). Attention of these group of RiPPs has spurred from their inherent structural stability arising from their characteristic circular peptide backbone. Cyclotides are perhaps the most characterized member of circular peptides. They are of plant-origin occurring in a large number of plants families (Craik, 2009). However, circular bacteriocins slightly differ from cyclotides as they do not possess intra-molecular disulfide cross-linkages. Unlike cyclotides, which are synthesized as precursor peptides with extensions at both terminal ends, circular bacteriocins only possess extensions at the N-terminal end known as the leader peptide. Due to these disparities, it is believed that their biosynthetic mechanisms are different (Masuda et al., 2012b).

To date, there are only 14 circular bacteriocins that have so far been characterized. Enterocin AS-48, the representative of the group was first reported in 1986 (Galvez et al., 1986), but its circular structure was only known in 1994 (Samyn et al., 1994). Since then, more circular bacteriocins have been discovered from different bacterial species: gassericin A (Kawai et al., 1998), circularin A (Kemperman et al., 2003b), butyrivibriocin AR10 (Kalmokoff et al., 2003), uberolysin (Wirawan et al., 2007), carnocyclin A (Martin-Visscher et al., 2008), lactocyclicin Q (Sawa et al., 2009), garvicin ML (Borrero et al., 2011), leucocyclicin Q (Masuda et al., 2011), amylocyclicin (Scholz et al., 2014), acidocin B [previously reported to be a linear bacteriocin (ten Brink et al., 1994), but was later known to have a circular structure (Acedo et al., 2015)], enterocin NKR-5-3B (Himeno et al., 2015), pumilarin (van Heel et al., 2017), and most recently, plantaricyclin A (Borrero et al., 2018) (**Table 1**).

**Table 1:** Relevant characteristics of circular bacteriocins.

Bacteriocin	Length (AA)		MW *^a^* (Da)	pI *^b^*	Net charge *^b^*	Hydrophobicity *^c^* (GRAVY Index) *^d^*	Producer strain	Reference
	Leader	Core peptide						
**Group i**								
Enterocin AS-48	35	70	7149.48	10.83	+6	0.539	*Enterococcus faecalis* S-48	Samyn et al., 1994
Circularin A	3	69	6770.99	11.13	+4	1.007	*Clostridium beijerinckii* ATCC 25752	Kemperman et al., 2003b
Uberolysin	6	70	7048.22	10.17	+3	0.937	*Streptococcus uberis* 42	Wirawan et al., 2007
Carnocyclin A	4	60	5861.98	10.70	+4	1.058	*Carnobacterium maltaromaticum* UAL307	Martin-Visscher et al., 2008
Lactocyclicin Q	2	61	6060.09	10.50	+4	0.826	*Lactococcus* sp. QU 12	Sawa et al., 2009
Garvicin ML	3	60	6007.21	10.85	+5	0.887	*Lc. garvieae* DCC43	Borrero et al., 2011
Leucocyclicin Q	2	61	6115.16	10.33	+3	0.744	*Leuconostoc mesenteroides* TK41401	Masuda et al., 2011
Amylocyclicin	48	64	6381.55	10.41	+5	0.850	*Bacillus amyloliquefaciens* FZB42	Scholz et al., 2014
Enterocin NKR-5-3B	23	64	6316.46	10.47	+5	0.953	*E. faecium* NKR-5-3	Himeno et al., 2015
Pumilarin	38	70	7087.35	10.72	+5	0.579	*B. pumilus* B4107	van Heel et al., 2017
**Group ii**								
Gassericin A	33	58	5653.54	7.54	+1	0.997	*Lactobacillus gasseri* LA39	Kawai et al., 1998
Butyrivibriocin AR10	22	58	5981.90	3.88	−2	1.002	*Butyrivibrio fibrisolvens* AR10	Kalmokoff et al., 2003
Acidocin B	33	58	5621.47	7.54	+1	1.036	*Lb. acidophilus* M46	Acedo et al., 2015
Plantaricyclin A	33	58	5570.47	9.53	+2	1.057	*Lb. plantarum* NI326	Borrero et al., 2018

*^a^Calculated molecular weight of the circular form of the bacteriocin using GeneScript^®^ Peptide Property Tool. ^b^Predicted for the circular form of the bacteriocin using GeneScript^®^ Peptide Property Tool. ^c^Predicted for the linear form of the bacteriocin using ExPaSy ProtParam Tool. ^d^Grand Average of Hydropathy.*

Previously, a circular antimicrobial peptide known as subtilosin A, produced by *Bacillus subtilis*, was initially considered to be a circular bacteriocin (Kawulka et al., 2003, 2004). However, due to its atypical characteristics such as its being negatively charged, having intra-molecular disulfide bridges, and being significantly smaller than the other circular bacteriocins, it has been suggested that subtilosin A should be reclassified as a new class of bacteriocin known as sactibiotics (Alvarez-Sieiro et al., 2016).

**FIGURE 1:**
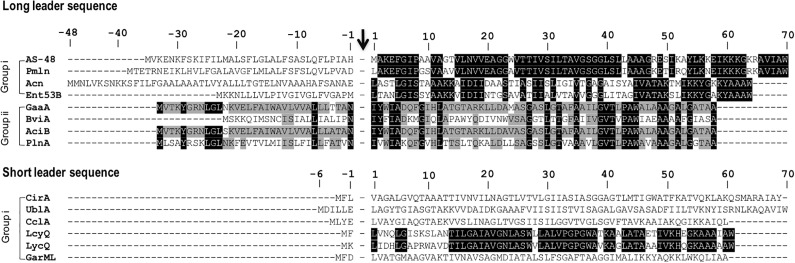
Primary structures of circular bacteriocin precursor peptides. Amino acid positions are numbered and the point of cleavage between the leader peptide and the core peptide is indicated by an arrow. Enterocin AS-48 (AS-48), pumilarin (Pmln), amylocyclicin (Acn), enterocin NKR-5-3B (Ent53B), gassericin A (GaaA), butyrivibriocin AR10 (BviA), acidocin B (AciB), and plantaricyclin A (PlnA) are members of the circular bacteriocins with long leader sequence, whereas circularin A (CirA), uberolysin A (UblA), carnocyclin A (CclA), leucocyclicin Q (LcyQ), lactocyclicin Q (LycQ), and garvicin ML (GarML) belong to circular bacteriocins with short leader sequence. Sub-grouping based on their biochemical features are also indicated as groups i and ii. Circular bacteriocins belonging to group ii showed high sequence homology. Whereas overall sequence comparison of group i bacteriocins showed very limited sequence homology, however, pairwise comparison between, AS-48 and Pmln, Acn and Ent53B, and LcyQ and LycQ showed very high sequence identity. Intensity (black to gray) of the highlighted residue indicates strong conservation of their residues.

The sub-classification among circular bacteriocins appears to be difficult since they display very little sequence similarity. Nevertheless, circular bacteriocins share a common structural fold known as the saposin fold (Martin-Visscher et al., 2009) and these peptides all possess ultra-stability against thermal stress, pH variation, and are generally resistant to degradation by many proteolytic enzymes. The compact and circular nature of their structures are thought to be the main factor contributing to their ultra-stability (Montalbán-Lopéz et al., 2008; Craik et al., 2010). On the basis on these similarities, a sub-classification based mainly on their biochemical characteristics have been suggested. In this scheme, circular bacteriocins are sub-divided into two sub-groups. Group i peptides are highly cationic and have high isoelectric points (pI > 9), while group ii peptides are highly hydrophobic, as they contain more acidic residues, and have predicted pI relatively lower than group i (pI < 7). Group i includes enterocin AS-48, circularin A, uberolysin, carnocyclin A, lactocyclicin Q, garvicin ML, leucocyclicin Q, amylocyclicin, enterocin NKR-5-3B, and pumilarin. Whereas group ii circular bacteriocins only comprise four members but are highly homologous, gassericin A, butyrivibriocin AR10, acidocin B, and very recently plantaricyclin A (**Table 1**). However, in the context on their possible biosynthetic mechanisms wherein the leader peptides are presumed to playing a key role, a different clustering scheme based on the length of their leader peptides has also been suggested (**Figure 1**) (Masuda et al., 2012b). It is worth noting that all group ii circular bacteriocins possess long leader sequences and showed considerable homology in their sequences. They have considerably lower isoelectric points (pI) and net charge but are more hydrophobic than most members of group i (**Table 1**).

### Genetics of Circular Bacteriocins

**FIGURE 2:**
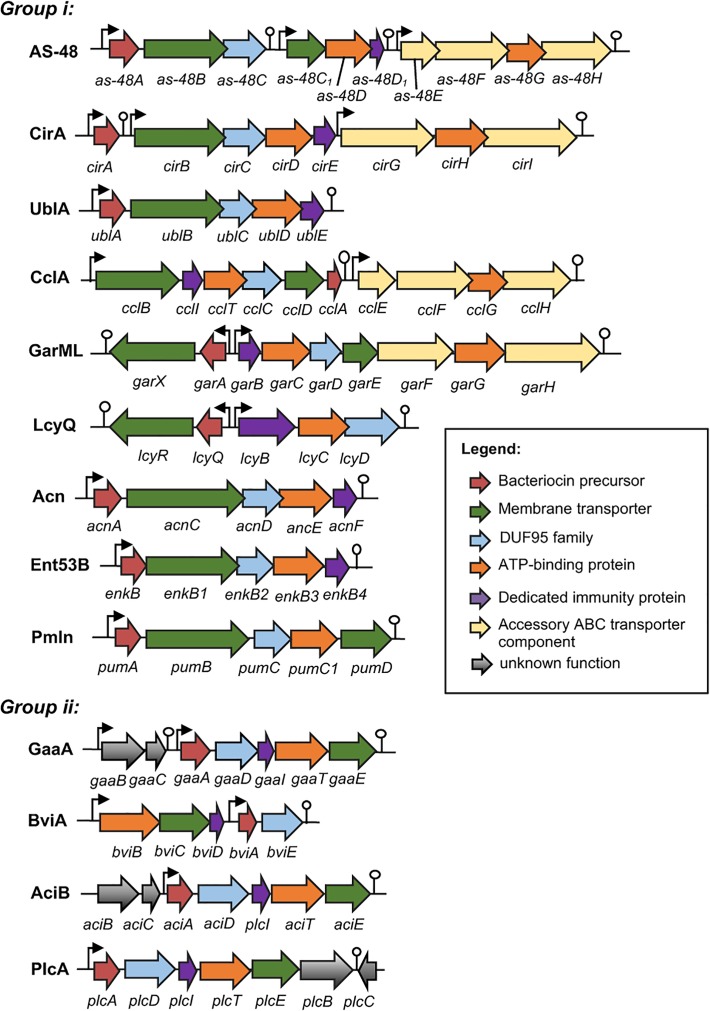
Biosynthetic gene cluster of circular bacteriocins. Genes are color coded based on their known and putative functions as indicated. Abbreviation of bacteriocins are shown in the legend of **Figure 1**. The bent arrows and lollipop symbols represent putative promoters and putative terminators, respectively. Figures are not drawn in scale.

Just as in the case of other bacteriocins, the genetic determinants of circular bacteriocins are either encoded chromosomally or on plasmids. The gene clusters involved in the biosynthesis of enterocin AS-48 (Martínez-Bueno et al., 1998), circularin A (Kemperman et al., 2003a), butyrivibriocin AR10 (Kalmokoff et al., 2003), uberolysin (Wirawan et al., 2007), gassericin A (Ito et al., 2009), carnocyclin A (van Belkum et al., 2010), garvicin ML (Gabrielsen et al., 2012), leucocyclicin Q (Mu et al., 2014), amylocyclicin (Scholz et al., 2014), acidocin B (Acedo et al., 2015), enterocin NKR-5-3B (Perez et al., 2016), pumilarin (van Heel et al., 2017), and plantaricyclin A (Borrero et al., 2018) have already been described (**Figure 2**). For lactocyclicin Q only the sequence of its structure gene has been reported while the details of its biosynthetic gene cluster have not yet been described. Except the peptide product of the structure gene, majority of the putative proteins encoded in these gene clusters are hydrophobic and hence are presumed to be membrane associated. Interestingly, despite the low sequence homology among the putative proteins encoded by these genes, similarity on their architecture and deduced characteristics are still apparent. Interestingly, all biosynthetic gene clusters of circular bacteriocins possess a gene encoding a protein belonging to a conserved hypothetical protein superfamily known as SpoIIM [PF01944, formerly known as domain of unknown function 95 (DUF95) (**Figure 2**)].

The minimum biosynthetic gene cluster responsible for production and self-immunity of circular bacteriocins is composed of five to seven genes. Functional analysis of the gene clusters *as-48-ABCC*_1_*DD*_1_, *cirABCDE, gaaBCADITE, cclBITCDA, garAXBCDE, enkBB1B2B3B4, acnACDEF*, and *plcADITEB* revealed the indispensability of these genes in the productions and immunities of enterocin AS-48, circularin A, gassericin A, carnocyclin A, garvicin ML, enterocin NKR-5-3B, amylocyclicin and plantaricyclin A, respectively. However, the expressions of an accessory operon encoding an ABC transporter protein complex could enhance both production and immunity such as in the case of enterocin AS-48 and carnocyclin A. Higher bacteriocin production and immunity were observed when the genes *as-48EFGH* (Diaz et al., 2003) and *cclEGHI* (van Belkum et al., 2010) were co-expressed with their respective minimum biosynthetic gene clusters.

Although the biosynthetic gene clusters of butyrivibriocin AR10 (Kalmokoff et al., 2003), uberolysin (Wirawan et al., 2007), leucocyclicin Q (Mu et al., 2014), acidocin B (Acedo et al., 2015), and pumilarin (van Heel et al., 2017) have already been described, experimental verification has not yet been reported. The putative functions of the enzymes encoded in the respective gene clusters were only inferred based on their sequence homology to other reported circular bacteriocin biosynthetic enzymes.

The biosynthetic gene cluster of circular bacteriocins shares some basic features which include (i) a gene encoding the precursor peptide, (ii) a gene encoding a dedicated immunity protein, which is a small, cationic, and hydrophobic protein, (iii) genes encoding an ABC transporter composed of an ATPase and a membrane protein, which is thought to be involved in the bacteriocin secretion but can also confer partial immunity [such as the case of EnkB1 and EnkB3 of enterocin NKR-5-3B (Perez et al., 2016)], and (iv) a gene encoding a membrane protein belonging to the DUF95 protein family, which is presumed to be involved in the circularization of the bacteriocin despite the lack of direct experimental evidence (**Figure 2**).

### Biosynthesis of Circular Bacteriocins

Despite the identification of the gene clusters involved in the biosynthesis of circular bacteriocins, our understanding of their biosynthetic mechanism has progressed rather slowly compared to its plant-derived counterparts, cyclotides. Perhaps, the absence of a distinct motif in the leader peptide of circular bacteriocins largely contributes to the challenge in understanding its biosynthesis. The role of the leader peptide on bacteriocin biosynthesis, at least in other groups of bacteriocins, has already been established. The leader peptide of bacteriocins belonging to the same group shares a common motif that plays a role in the substrate-enzyme interaction processes (Oman and van der Donk, 2010). The leader peptides of type I lantibiotic bacteriocins have a conserve FNLD box and possess helical structures that are indispensable in their interaction with their cognate biosynthetic enzymes (Kuipers et al., 2004; Rink et al., 2007; Abts et al., 2013). Although also present in a wide range of peptide natural products, most class II non-lantibiotic bacteriocins possess a highly conserved double glycine motif at –1 and –2 positions in their leader peptides that is important in directing the secretion of the mature bacteriocins (van Belkum et al., 1997). However, leader peptides of circular bacteriocins are highly disparate both in length and sequences, thus making it difficult to predict their role in circular bacteriocin biosynthesis.

The leader peptides of circular bacteriocins range from 2 to 48 residues. It is increasingly becoming apparent that the leader peptides of circular bacteriocins do not function as recognition signal for its processing, i.e., cleavage, circularization, and secretion. In the case of garvicin ML, the leader peptide appears to be not required in these processes but still remain essential as a critical control checkpoint of its biosynthetic machinery (Gabrielsen et al., 2014b). Such function could be on the maintenance of an essential fold of the precursor peptide for its interaction with its processing enzymes. In the case of enterocin NKR-5-3B, any truncations in its leader peptide resulted in the blockage of the bacteriocin maturation. Whereas introduction of different point mutations into the leader peptide resulted in a variable bacteriocin production. It was thought that the mutations in the leader peptide altered the overall conformation of the precursor peptide that reduced or enhanced its ability to fit and interact with the substrate-binding cleft of its processing enzyme(s) (Perez et al., 2017). The same was observed when mutation at –1 position of the leader peptide was introduced to the enterocin AS-48 precursor which resulted in the interference of the maturation of the bacteriocin (Cebrián et al., 2010).

Clearly, the specific mechanisms of circular bacteriocin biosynthesis involving three events: cleavage of the leader peptide, circularization, and export to the extra-cellular space, have remained an enigma. It was previously thought that these events could be coupled reactions. However recently, it was suggested that cleavage of the leader peptide is independent and precede all these events in their biosynthesis. Mutants with some disabled biosynthetic genes (ΔgarBCDE and garX::pCG47) were only able to produce the precursor peptide and linear forms of garvicin ML suggesting that these knocked-out mutants lost the capacity for the circularization reaction (Gabrielsen et al., 2014b). Whereas, in the case of leucocyclicin Q, circularization and secretion have been shown to be separate processes (Mu et al., 2014). When lcyD, the biosynthetic gene encoding the enzyme belonging to DUF95, was knocked-out, the resulting mutant accumulated the mature circular bacteriocin inside the cells. In the case of AS-48, the linear form was also detected together with the circular form when the terminal residue Trp (W70) was mutated to Ala (Cebrián et al., 2010). However, this data was not enough to support the notion of the independence of the cleavage and circularization processes. The authors reasoned that the mutations could be a result of the dissociation of the leader peptide cleavage and circularization reactions.

But perhaps, the understanding the circularization process of these peptides is the most challenging. It is still a mystery how the enzyme(s) catalyze the reaction leading the “free” C-terminal end to be subject to attack by the amino group of the residue at the N-terminal end. Some clue may be found in the mature forms of circular bacteriocins. Interestingly, the ligation sites of all circular bacteriocins are located within a helical structure, a region composed mainly of hydrophobic residues. It was previously thought that these hydrophobic patches surrounding the ligation site are crucial for the interaction with their cognate biosynthetic enzyme(s) (Martin-Visscher et al., 2009). Indeed, extensive mutational analysis of the residue (Leu1) at position 1 of enterocin NKR-5-3B revealed that this consensus is crucial in the process of bacteriocin maturation. Only mutations with hydrophobic residues that have the propensity of promoting helical structure formation (Ala, Ile, Val, and Phe) successfully yielded the mature Ent53B derivatives; however, substitution with non-helix-promoting residues and non-hydrophobic residues failed to yield the mature bacteriocin (Perez et al., 2017). So far, only the circular bacteriocin enterocin AS-48 was subjected to a similar mutational analysis albeit limited to single amino acid substitution. Met at position 1 when mutated to Ala (Met1Ala) significantly affected the efficiency of bacteriocin maturation, while mutation at the C-terminal residue Trp with Ala (Trp70Ala) resulted in the production of both linear and circular species of the bacteriocin (Cebrián et al., 2010). It should be interesting to expand this analysis to other known circular bacteriocins to confirm the importance of the amino acid hydrophobicity and helix-forming capacity in the biosynthesis of circular bacteriocins.

With respect to the nature and type of enzyme(s) catalyzing the reactions during circular bacteriocin maturation, no clear evidence has so far been reported. Up to now, it is certain that the enzymes catalyzing these reactions are encoded in the gene clusters of four to five genes. In the case of enterocin NKR-5-3B, production of the mature circular bacteriocin was blocked when the phenotype is devoid of a single gene in its biosynthetic gene cluster. Analyses of the gene clusters of other circular bacteriocins showed similar pattern suggesting that no single protein is responsible for the maturation, particularly the circularization and secretion of these bacteriocins. It is highly possible that the enzymes encoded in these biosynthetic gene clusters would form a membrane-located complex and jointly process the three events in succession to complete the circular bacteriocin biosynthesis. However, the absence of any peptidase domain in the biosynthetic enzymes encoded by these genes punctuates the enigmatic event of the leader peptide cleavage. It has been suggested that the enzyme responsible catalyzing this reaction could be encoded elsewhere outside the biosynthetic gene cluster such as in the case of the chromosomally encoded signal peptidases (SPases) cleaving the N-terminal sequences of other bacterial circular peptides from *Escherichia coli* and *Agrobacterium tumefaciens* (Gabrielsen et al., 2014a). Proponents of the theory opined that this could be the reason for the difficulty in establishing a heterologous expression of the circular bacteriocin enterocin AS-48 outside the genus of its original producer (Fernández et al., 2007), as well as the apparent problem of the unstable heterologous expression of circularin A (Kemperman et al., 2003a). While it is possible that there could be other elements encoded outside the gene cluster needed for the circularization of circular bacteriocins, this notion still entails a more direct evidence. It was vaguely opined that the unstable heterologous expression system of circularin A in an enterococcal host was a consequence of a probable chromosomally encoded protease. It is worth noting that the success of the heterologous expression systems of these circular bacteriocins appear to show no consistent correlation between the phylogenetical distance of the original producer strain and the host. The producer and host of the successful heterologous expression system of circularin A is phylogenetically more distant than that of the host and producer strain of the unsuccessful inter-genus heterologous expression system of enterocin AS-48 (enterococcal host successfully functioned as a host for the clostridium-borne circularin A whereas a number of *Lactobacillus* strains failed as host for the enterococcal-borne enterocin AS-48). Whereas, in the case of enterocin NKR-5-3B, *Enterococcus* and *Lactobacillus* served as successful host for its heterologous expression (Perez et al., 2016).

With regards to the regulatory mechanisms of the production of circular bacteriocins, it has remained so far to be largely unknown. While genes encoding proteins that are commonly involved in the regulation of most bacteriocins such as histidine kinase and response regulator proteins were found in the vicinity of the biosynthetic gene clusters of some circular bacteriocins such as that of butyrivibriocin AR10, circularin, uberolysin, and enterocin NKR-5-3B, their cognate activator molecules have not been identified. Nonetheless, some hints pointing to the possible regulation by quorum sensing three component system are apparent (Bartholomae et al., 2017).

### Mode of Action of Circular Bacteriocins

The mechanism of the antimicrobial action of circular bacteriocins is based on its ability to directly interact with the bacterial cell membrane that would cause its permeation resulting to the leakage of ions, dissipation of membrane potential, and eventually, cell death. The common characteristics of circular bacteriocins of having high net positive charge is believed to facilitate their electrostatically driven interaction to the negatively charge bacterial membrane without requiring any receptor molecule. It was previously shown that enterocin AS-48 can cause molecular electroporation on target membranes by forming non-selective ion channels in the cytoplasmic membrane leading to the leakage of ions and low-molecular weight solutes (Gálvez et al., 1991; Gonzalez et al., 2000). While gassericin A induces cell death through the leakage of potassium ions resulting from the permeation of the membranes of sensitive cells (Kawai et al., 2004). Whereas carnocyclin A has been shown to form anion selective and voltage dependent pores on target membranes (Gong et al., 2009).

The identification of two different oligomeric states of enterocin AS-48 provided the detailed mechanism on its membrane interaction. X-ray crystallography analysis revealed that enterocin AS-48 exists in water-soluble dimeric form (DF-I) that would rearrange into its membrane-bound dimeric form (DF-II) facilitating its insertion into the membrane (Sánchez-Barrena et al., 2003). However, recently this mechanism was modified after their biochemical and structural studies of some mutated enterocin AS-48. In the modified mechanism, the dimeric enterocin AS-48 is believed to dissociate into its amphiphilic and monomeric bacteriocin form as it makes contact with the membrane periphery due to the acidic environment of the membrane. The monomeric bacteriocin would then establish both electrostatic and hydrophobic interactions after it inserts into the membrane. The accumulation of the bacteriocin molecules in the membrane would cause its permeation and ultimately cell death (Cebrián et al., 2015).

Although the mode of action of most circular bacteriocins has not yet been examined, at least in the case of enterocin AS-48, carnocyclin A, and gassericin A, it has been established that these circular bacteriocins do not require a receptor molecule for its antimicrobial action (Gálvez et al., 1991; Kawai et al., 2004; Gong et al., 2009). Additionally, enterocin NKR-5-3B was also shown to have strong affinity for negatively charged membranes illustrating its capacity to non-selectively bind to target membrane without the need of a receptor molecule (Himeno et al., 2015). However, it has been suggested that circular bacteriocins might have an alternate concentration-dependent mode of action, similar to that of nisin A. It has been shown that the maltose ABC transporter complex is a possible receptor molecule for the antimicrobial action of garvicin ML. The expression of this sugar uptake system facilitated the sensitivity to the bacteriocin while its absence caused resistance (Gabrielsen et al., 2012). This finding contradicted the previous understanding on the non-selective membrane interactions of circular bacteriocins. The authors argued that this could be due to the significantly higher bacteriocin concentrations used for the membrane interaction assays than their actual *in vivo* inhibitory concentrations (Gabrielsen et al., 2014a). They hypothesize further that circular bacteriocins could possess a dual-mode of action that is concentration-dependent such as in the case of the lantibiotic nisin A. Indeed, nisin A was shown to exhibit two concentration-dependent modes of action. At high concentration, nisin A molecule triggers pore formation in the membrane thereby inducing death. While at low concentrations, it kills target bacteria by inhibiting cell wall synthesis. However, it is worth noting that in both modes of action, the interaction of nisin A molecule with lipid II as its receptor molecule remained essential (Breukink et al., 1999; Wiedemann et al., 2001).

## Leaderless Bacteriocins

As discussed above, bacteriocins are synthesized as an inactive precursor peptide having N-terminal extensions, known as leader peptides, attached to the C-terminal core peptide moiety. However, some bacteriocins are atypical since they are synthesized without an N-terminal extension, hence the name “leaderless bacteriocins.” The mystery surrounding leaderless bacteriocins abounds since it has long been acknowledged that leader peptides are indispensable in bacteriocin biosynthesis. The functions of the leader peptide include: (i) serves as a recognition site for the biosynthetic enzymes, (ii) protects the producer strain from the bacteriocin’s inhibitory activity, and (iii) ensure a suitable conformation essential for the enzyme–substrate interaction (Klaenhammer, 1993; van der Meer et al., 1994; Oman and van der Donk, 2010). Questions regarding the basic biology of their biosynthesis remained unanswered. Leaderless bacteriocin do not undergo any post-translational modifications or processing and become active soon after translation. Hence, it is a mystery how the producer cell protects itself from its own bacteriocin before they are secreted outside the cell. Do the producer cells possess unique immunity systems that confer both intra- and extracellular protection? After all, it has been shown that overexpression of lacticin Q (in the absence of its biosynthetic machinery) causes intracellular toxicity that can suppress the growth of the host cells (Iwatani et al., 2012). It is also interesting how these bacteriocins are secreted to the extracellular space as they lack transport signal domains typical to most bacteriocins. Undeniably, leaderless bacteriocins appear to be the most enigmatic and poorly understood group of bacteriocins.

Interestingly, one common feature of most leaderless bacteriocins is the presence of formylated methionine at the N-terminal. Although its significance in the antimicrobial and/or in the biosynthetic process still remains unknown. However, the removal of *N*-formyl-methionine after cyanogen bromide treatment did not have any negative effect on the bioactivities of lacticins Q and Z. Moreover, *in vitro*-synthesized lacticin Q with non-formyl methionine at its N-terminal still exhibited comparable antimicrobial activity (unpublished data). Furthermore, lacticin Q and aureocin A53 obtained from overexpression in *E. coli* BL21 (DE3) yielding a non-formylated N-terminal methionine peptides both demonstrated intact antimicrobial activities (Acedo et al., 2016). Hence, the formylation of methionine at the N-terminal may not have significant role in the bioactivity of leaderless bacteriocins.

The first reported leaderless bacteriocin, enterocin L50, is composed of two peptides (enterocin L50A and L50B) that exhibit synergistic activity (Cintas et al., 1998). However, they should not be confused with the two-peptide bacteriocins (a group consisting of two complementary peptides), since they possess distinct characteristics that set them apart from these bacteriocins (i.e., each peptide has significant activity alone and are being synthesized without a leader peptide). Recently, there has been a growing number of reports describing leaderless bacteriocins of varying characteristics, suggesting that leaderless bacteriocins could be a huge group of antimicrobial peptides. Leaderless bacteriocins that are single peptide, two-peptide and multi-peptide (3–4 peptides) bacteriocins have already been described (**Table 2**). Leaderless bacteriocins have comparable pI values and net cationic charge with class i circular bacteriocins but are significantly higher than of group ii circular bacteriocins. Most notably, leaderless bacteriocins are significantly less hydrophobic than circular bacteriocins (**Tables 1**, **2**).

**Table 2:** Relevant characteristics of different leaderless bacteriocins.

Bacteriocin	Length (AA)	MW *^a^* (Da)	pI *^b^*	Net charge *^b^*	Hydrophobicity *^c^* (GRAVY Index) *^d^*	Producer strain	Reference
**Single peptide**							
Enterocin Q	34	3979.73	9.78	+4	−0.359	*E. faecium* L50	Cintas et al., 2000
Aureocin A53	51	6,012.19	10.73	+8	−0.08	*Staphylococcus aureus* A53	Netz et al., 2002
BHT-B	44	5193.06	10.57	+4	0.241	*S. rattus* strain BHT	Hyink et al., 2005
LsbB	30	3437.99	10.75	+6	−0.683	*Lc. lactis* subsp. *lactis* BGMN1-5	Gajic et al., 2003
Lacticin Q	53	5926.06	10.84	+6	0.300	*Lc. lactis* QU 5	Fujita et al., 2007
Lacticin Z	53	5968.10	10.63	+5	0.28	*Lc. lactis* QU 14	Iwatani et al., 2007
Weisselicin Y	42	4923.68	10.36	+5	−0.090	*Weissella hellenica* QU 13	Masuda et al., 2012a
Weissellicin M	43	4966.85	10.39	+5	0.037	*W. hellenica* QU 13	Masuda et al., 2012a
Enterocin EJ97	44	5350.29	10.39	+6	−0.589	*E. faecalis* EJ97	Sánchez-Hidalgo et al., 2003
Enterocin K1	37	4592.37	10.24	+6	−0.700	*E. faecium* EnGen0026	Ovchinnikov et al., 2017
Epidermicin NI01	51	6072.27	10.95	+9	−0.020	*S. epidermidis* strain 224	Sandiford and Upton, 2012
Lactolisterin BU	43	5163.02	10.72	+5	−0.151	*Lc. lactis* subsp. *lactis* bv. *diacetylactis* BGBU1-4	Lozo et al., 2017
**Two-peptide**							
Enterocin L50						*E. faecalis* L50	Cintas et al., 1998
L50A	44	5218.35	10.68	+7	0.202		
L50B	43	5206.25	10.95	+7	−0.144		
Enterocin MR10 (DD14)*^e^* (Ent7)*^f^*						*E. faecalis* MRR 10-3; 14; 710C	Martín-Platero et al., 2006; Liu et al., 2011; Caly et al., 2017
MR10A (DD14A) (Ent7A)	44	5204.32	10.68	+7	0.202		
MR10B (DD14B) (Ent7B)	43	5210.24	10.95	+7	−0.109		
**Multi-peptide**							
Aureocin A70						*S. aureus* A70	Netz et al., 2001
A70A	31	2952.53	10.98	+4	0.529		
A70B	30	2825.34	10.87	+4	0.707		
A70C	31	2982.56	10.87	+5	0.552		
A70D	31	3114.76	10.7	+4	0.632		
Garvicin KS						*Lc. garvieae* KS1546	Ovchinnikov et al., 2016
KS-A	34	3481.14	10.98	+4	0.403		
KS-B	34	3188.84	11.02	+5	0.691		
KS-C	32	3127.76	10.98	+4	0.588		
Cereucin X*^g^*						*Bacillus cereus* BAG2O-1	Ovchinnikov et al., 2016
X-A	27	2972.48	10.33	+2	0.004		
X-B	29	3165.63	9.92	+3	−0.186		
X-C	30	2826.25	10.64	+4	0.520		
Cereucin H*^g^*						*B. cereus* HuA2-4	Ovchinnikov et al., 2016
H-A	26	2876.39	10.03	+2	0.154		
H-B	30	3170.70	10.50	+2	0.233		
H-C	30	2867.42	10.87	+4	0.667		
H-D	30	3018.63	10.86	+4	0.620		
Cereucin V*^g^*						*B. cereus* VD148	Ovchinnikov et al., 2016
V-A	30	3142.69	11.08	+3	0.373		
V-B	30	2857.41	10.87	+4	0.650		
V-C	31	3004.52	10.88	+3	0.690		

*^a^Calculated molecular weight of its N-terminal formylated methionine form using GeneScript^®^ Peptide Property Tool. ^b^Predicted for its N-terminal formylated methionine form using GeneScript^®^ Peptide Property Tool. ^c^Predicted for non-formylated methionine form using ExPaSy ProtParam Tool. ^d^Grand Average of Hydropathy. ^e,f^Produced by a different strain E. faecalis 14^e^ and E. faecalis 710C^f^. ^g^Putative multi-peptide leaderless bacteriocins based on genetic sequence homology.*

**FIGURE 3:**
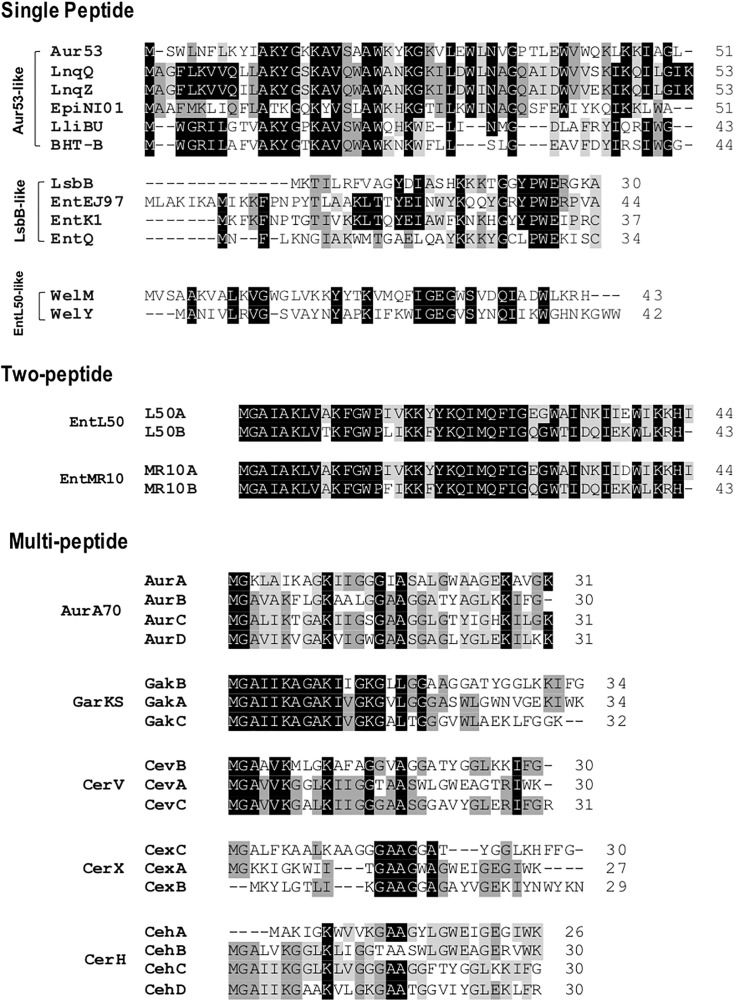
Primary structures of leaderless bacteriocins. Leaderless bacteriocins are clustered based on the number of their component peptides; single-peptide, two-peptide, and multi-peptide. Single peptide group is composed of aureocin A53 (Aur53), lacticins Q and Z (LnqQ and Z), epidermicin NI01 (EpiNI01), lactolisterin BU (LliBU), BHT-B, LsbB, enterocins EJ97, K1, and Q (EntEJ97, EntK1, and EntQ, respectively), and weissellicins M and Y (WelM and Y). Two-peptide group is composed of enterocins L50 (L50A and B) and enterocins MR10 (EntMR10A and B). Whereas multi-peptide group is composed of aureocin A70 (AurA, B, C, and D), garvicin KS (GakA, B, and C), cereucin V (Cev A, B, and C), cereucin X (cexA, B, and C), and cereucin H (CehA, B, C, and D). Sub-clustering based on their sequence homology is also indicated; aur53-like, LsbB-like, and entL50-like. Intensity (black to gray) of the highlighted residue indicates strong conservation of their residues.

Leaderless bacteriocins display high sequence homology to most members within their sub-groups (**Figure 3**). The aureocin A53-like single leaderless bacteriocins are highly cationic and are Trp-rich peptides. The Trp residues of these group are believed to play a key role in their antimicrobial action as it facilitates their interaction with the bacterial membranes (Fimland et al., 2002). Members of this sub-group are aureocin A53, lacticins Q and Z, epidermicin NI01, lactolisterin BU, and BHT-B. These bacteriocins exhibit highly conserved N-terminal region (Ala^11^ to Leu^29^ with aureocin A53 as reference) and relatively variable C-terminal region (**Figure 3**). Whereas, LsbB-like sub-group, with members LsbB, enterocins EJ97, K1, and Q, show relatively conserved C-terminal region (Lys^17^ to Glu^26^ with LsbB as reference) and a less conserved N-terminal region (**Figure 3**). On the other hand, although weissellicins Y and M belong to single peptide leaderless bacteriocins (for the reason that they do not show synergistic activity and encoded in separate operons) they exhibit high similarity to two-peptide leaderless bacteriocins (Masuda et al., 2012a). Two-peptide leaderless bacteriocins so far has two members; enterocin L50s (L50A and B) and MR10 (MR10A and B) (**Figure 3**). Two other bacteriocins identical to enterocin MR10 produced by other strains have been reported with other names; enterocins DD14 (DD14A and B) and enterocin 7 (Ent7A and B) (**Table 2**).

Recently, a novel sub-group of multi-peptide (three to four peptide) leaderless bacteriocin was described (Ovchinnikov et al., 2016). Although the four-peptide leaderless bacteriocin, aureocin A70, was reported as early as 2001, other bacteriocins with such similar feature were not described until this report. The genetic loci of these multi-peptide bacteriocins showed conserved genetic organization, including being located adjacent to conserved genetic determinants. Thus far, this sub group has five members; aureocin A70 (AurA, B, C, and D), garvicin KS (GakA, B, and C), cereucin V (CevA, B, and C), cereucin X (CexA, B, and C), and cereucin H (CehA, B, C, and D) (**Table 2** and **Figure 3**). Sequence homology analysis of garvicin KS facilitated the discovery of cereucins V, X, and H from unannotated genes of some *Bacillus cereus* strains found in genomic database. The antimicrobial activity of all these bacteriocins was confirmed with synthetic peptides and all were shown to have broad spectrum antimicrobial spectra (Ovchinnikov et al., 2016).

### Genetics and Biosynthesis of Leaderless Bacteriocins

**FIGURE 4:**
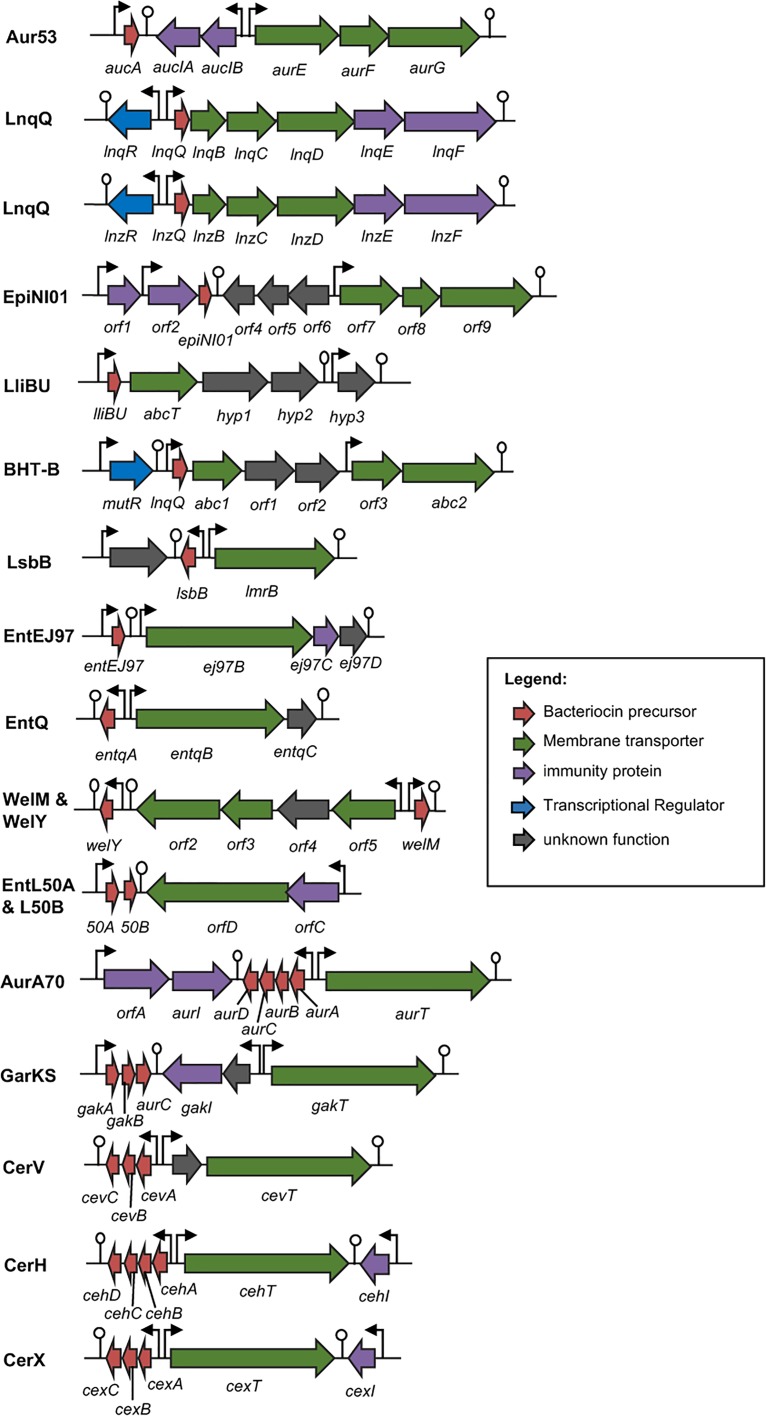
Biosynthetic gene cluster of leaderless bacteriocins. Genes are color coded based on their known and putative functions as indicated. Abbreviation of bacteriocins are shown in the legend of **Figure 3**. The bent arrows and lollipop symbols represent putative promoters and putative terminators, respectively. Figures are not drawn in scale.

To date, only very little is known on the biosynthetic mechanisms of leaderless bacteriocins. Although the genetic determinants of most leaderless bacteriocins have already been reported (**Figure 4**), only in the case of lacticins Q and Z (Iwatani et al., 2012, 2013), aureocin A53 (Nascimento Jdos et al., 2012) and aureocin A70 (Netz et al., 2001; Coelho et al., 2014) that the genes responsible for both their secretion and immunity have been experimentally confirmed. While the ABC-type multi-drug resistance transporter protein, LmrB, has been previously reported to be involved in both secretion and immunity of the leaderless bacteriocin, LsbB, and a non-leaderless bacteriocin, LsbA (Gajic et al., 2003). However, an apparent common mechanism or consensus of their biosynthesis has remained elusive. Nonetheless, reports describing the putative bacteriocin operons outlining the genetic organizations of most leaderless bacteriocins are already available. The structure genes of the enterocin L50 (L50A and B) were encoded on a 50-kb plasmid, pCIZ1 (Cintas et al., 1998). Whereas, enterocin Q, produced by strain L50 together with enterocin L50, is encoded on a 7.4-kb plasmid pCIZ2 (Cintas et al., 2000; Criado et al., 2006a). Although enterocin L50 has already been successfully expressed in at least two yeast expression systems, their production was through the general secretory pathways of the host rather than via its own genetics determinants (Basanta et al., 2009, 2010).

Additionally, the structure genes and the genes encoding the putative biosynthetic enzymes of weissellicin Y and M and enterocin MR10 (MR10A and B) seem to be encoded in the chromosomal DNA of its producer strains *Weissella hellenica* QU 13 (Masuda et al., 2012a) and *Enterococcus faecalis* MRR10-3 (Martín-Platero et al., 2006), respectively. Whereas the plasmid-encoded genetic determinants for the leaderless bacteriocins enterocin EJ97 (Sánchez-Hidalgo et al., 2003), lactolisterin BU (Lozo et al., 2017), garvicin KS (Ovchinnikov et al., 2016), cereucin H, V, and X (Ovchinnikov et al., 2016), and epidermicin NI01 (Sandiford and Upton, 2012) have also been described. Although, the specific functions of the gene products encoded in these putative biosynthetic genes have remained unconfirmed.

With respect to the regulation of their production, it is apparent that there is no common mechanism existing among leaderless bacteriocins. In the case of lacticin Q, it was recently shown that LnqR, a protein belonging to the TetR family of transcriptional regulators, positively regulates the transcription of its respective biosynthetic genes *lnqBCDEF* and consequently its production (Iwatani et al., 2016). While in the case of aureocin A70, a complex mechanism involving a protein with a helix-turn-helix (HTH) AurR, an alternative transcription factor σ^B^, and a phage regulator ϕ11, regulates its production (Coelho et al., 2016). Whereas in the case of the *W. hellenica* QU 13, the producer of weissellicins Y and M, it was demonstrated that its bacteriocin production has a nutrition-adaptive control. The presence of thiamine although enhanced its growth, significantly decreased the production of weissellicin Y but no effect on weissellicin M production (Masuda et al., 2016). While the productions of enterocin L50 (enterocin L50A and B) and enterocin Q in the multiple bacteriocin producing strain *E. faecium* L50 were shown to be temperature-regulated. Enterocins L50A and B were synthesized in the range of 16°C–32°C, but the production became negligible beyond 37°C, whereas enterocin Q was produced at a wider temperature range of 16°C–47°C (Criado et al., 2006b).

### Mode of Action of Leaderless Bacteriocins

Generally, leaderless bacteriocins have been shown to not require a receptor molecule for its killing mechanisms. Lacticin Q has perhaps the most characterized mode of action among leaderless bacteriocins. Lacticin Q exhibits a very strong bioactivity at nanomolar levels against a number of Gram-positive bacteria including a number of *Bacillus* sp., *Lactobacillus* sp., *Enterococcus* sp., *Lactococcus* sp., and *Staphylococcus aureus* (Fujita et al., 2007). Lacticin Q has been shown to form huge toroidal pore (HTP) ranging 4.6–6.6 nm in size, enough to cause leakage of large intracellular components such as proteins and other small cytosolic components such as ions and ATP, thereby inducing cell death. The HTP formation mechanism starts with the electrostatic interaction of the cationic lacticin Q molecules and the negatively charged membranes. The rapid binding of lacticin Q to the phospholipid bilayer membranes results in the formation of HTPs coupled with lipid flip-flop. Intracellular components then escape from these pores, leading to cell death. The pores formed in the membrane are short-lived because these HTPs close back as the lacticin Q molecules translocates itself beneath the cell membrane (Yoneyama et al., 2009). However, in spite of the non-requirement of a docking molecule for its mode of action, lacticin Q’s interaction with target bacteria are still highly dependent on the physiological features of their cell membrane thus causing its selective inhibitory action (Yoneyama et al., 2011). The antimicrobial activity of lacticin Q is surprisingly selective and variable within species, and in some cases, within strains of its sensitive bacteria. This variability is thought to be caused by both its affinity to the bacterial membrane (leading to the formation of HTPs) as well as the strains’ capacity to scavenge the hydroxyl radicals, which differs from each strain. The accumulation of the toxic hydroxyl radicals serves as the final antimicrobial mechanism of lacticin Q (Li et al., 2013).

Whereas in the case of the leaderless aureocin A53, it was also shown to permeate the membranes of its target bacteria but do not form discrete pores (Netz et al., 2002). However, it is interesting that aureocin A53 molecules exhibited stronger interaction with neutral membrane than negatively charged lipids. This observation undermined the role of the negatively charged lipids in the initial peptide–membrane electrostatic interaction mechanism of its mode of action. In any case, this leaderless bacteriocin still exhibited a broad range of bioactivity against a number of bacterial strains including notable pathogens such as vancomycin resistant *Enterococcus*, *Listeria innocua*, and *S. aureus* (Netz et al., 2002).

Contrary to the general understanding that leaderless bacteriocins do not require a receptor molecule for its mode of action, studies on the leaderless bacteriocin, LsbB, isolated from *L. lactis* subsp. *lactis* BGMN1-5, identified a zinc-dependent membrane metallopeptidase, YvjB, as its receptor molecule (Uzelac et al., 2013). The outmost 8-amino acid sequence at the C-terminal end of LsbB has been shown to contain the receptor binding domain (Ovchinnikov et al., 2014). Further analyses have shown that the receptor-binding domain of LsbB interacts with the highly conserved Tyr^356^ and Ala^353^ residues at the third transmembrane domain of YvjB (Miljkovic et al., 2016). They further hypothesized that this killing mechanism is common among LsbB-related leaderless bacteriocins such as enterocins Q, K1, and EJ97 (Ovchinnikov et al., 2014, 2017).

## Structural Resemblance Between Circular and Leaderless Bacteriocins

**FIGURE 5:**
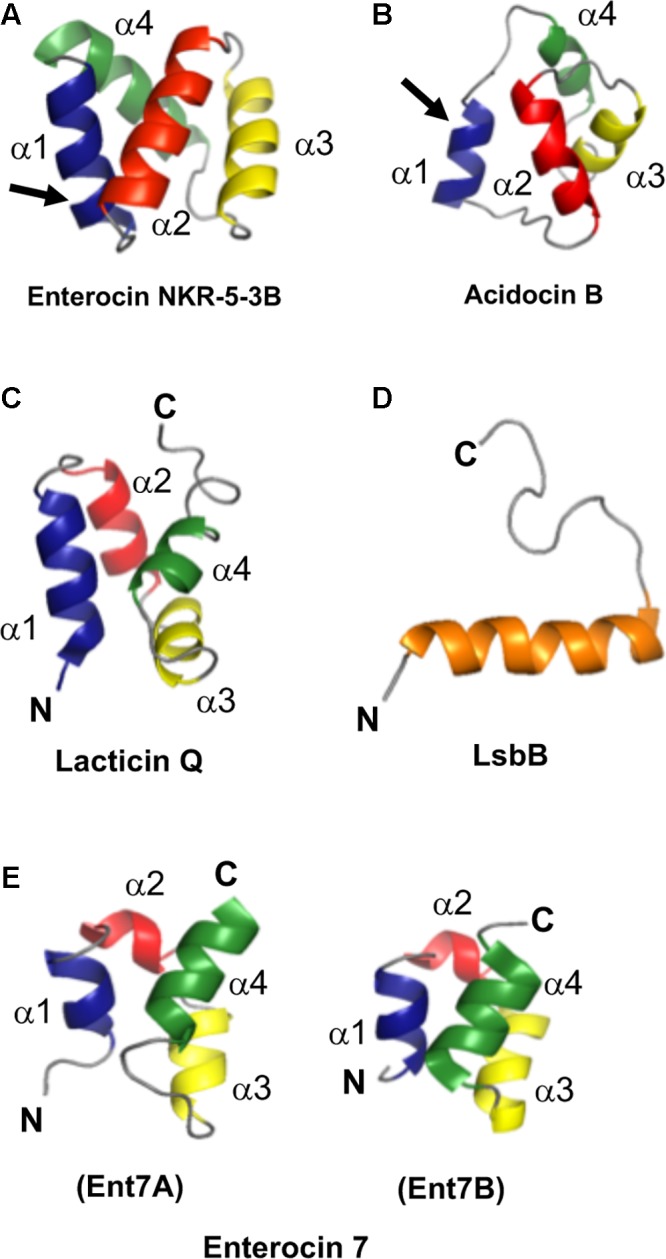
Cartoon representation of the structures of the representative sub-groups of circular and leaderless bacteriocins. **(A)** enterocin NKR-5-3B represents the saposin fold exhibiting circular bacteriocins or group i, **(B)** acidocin B represents the helical bundle structure exhibiting circular bacteriocins or group ii, **(C)** lacticin Q represents the saposin fold exhibiting single peptide leaderless bacteriocins, **(D)** LsbB represents the single helical structure exhibiting single peptide leaderless bacteriocins, and **(E)** enterocin 7, composed of enterocin 7A and 7B, represents the saposin fold exhibiting two-peptide leaderless bacteriocins. N- and C-terminal ligation site of the circular bacteriocins is indicated by arrow whereas the terminal ends of leaderless bacteriocins are indicated by letters N and C. In order to depict directionality, helices of each bacteriocins are numbered accordingly. Images are rendered using PyMol Molecular Graphics System.

As mentioned above, a common structural motif known as saposin fold exists among most circular bacteriocins, particularly the circular bacteriocins belonging to group i. Saposins are a group of four proteins produced in humans and are involved in sphingolipid catabolism (Vaccaro et al., 1999). The so-called saposin fold refers to the structural confirmation by four or five amphipathic α-helices arranged in a distinctly compact architecture forming a small hydrophobic core (Liepinsh et al., 1997; Bruhn, 2005; Ahn et al., 2006). Although the structure of group ii circular bacteriocins showed some differences in the overall folding of the saposin-like exhibiting group i circular bacteriocins, they still display some degree of similarity. Group ii circular bacteriocins possess distinct helical-bundle structures forming similar directionality with that of group i circular bacteriocins (**Figure 5B**). The overall fold of the amphipathic helices of both groups resulted in the formation of a hydrophobic core as a consequence of the packing of the hydrophobic residues (**Figures 5A,B**) (Towle and Vederas, 2017).

**FIGURE 6:**
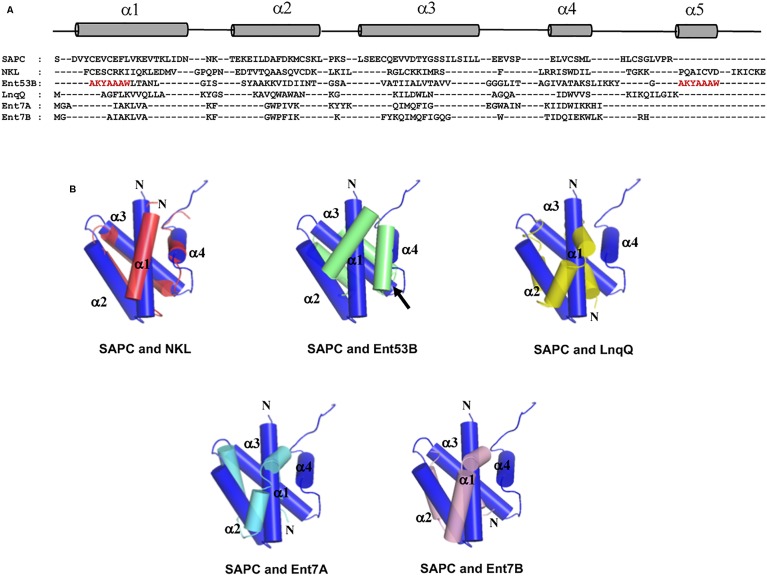
Sequence and structure alignment of representative peptides of saposin fold exhibiting bacteriocins; enterocin NKR-5-3B (Ent53B), lacticin Q (LnqQ), enterocin 7 (Ent7A and 7B), saposin and saposin-like peptide. **(A)** Sequence alignment highlighting the helical and coil regions of these peptides as represented by the cylinder and line symbols, respectively, **(B)** pair-wise superimposition of the structure of saposin C (SAPC; blue) with the structures of NK-lysin (NKL; red), enterocin NKR-5-3B (Ent53B; green), lacticin Q (LnqQ; yellow–green), enterocin 7 (Ent7A; cyan and 7B; pink). The helices are numbered accordingly with SAPC as the reference. The N-termini of the peptides and the ligation site of Ent53B are indicated by the letter N and an arrow, respectively. In the case of Ent53B the region in the “fifth helix” is repeated in the first helix (amino acid sequence are colored red) due to the N- and C-terminal ligation. Images are generated using PyMol Molecular Graphics System.

**FIGURE 7:**
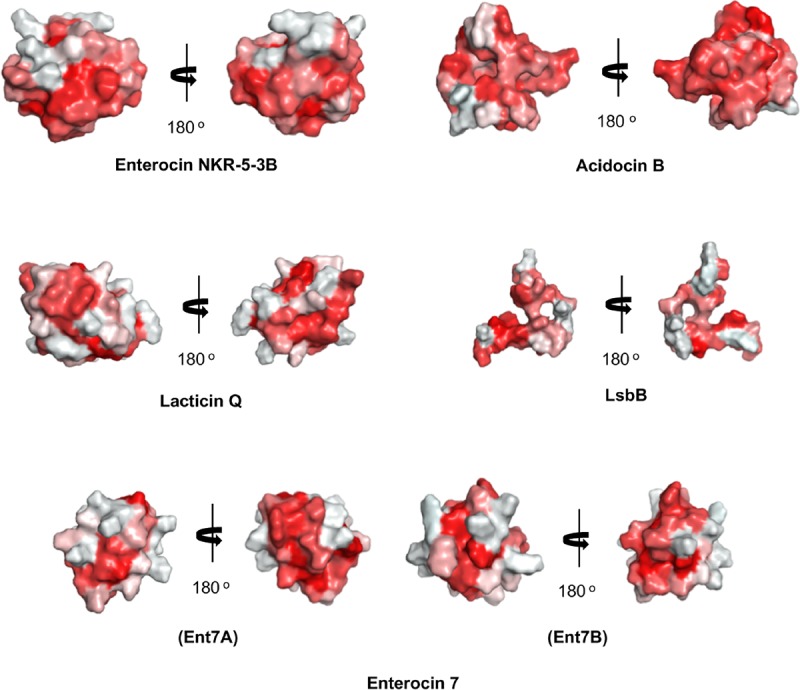
Hydrophobic surface maps of representative sub-groups of circular and leaderless bacteriocins. Enterocin NKR-5-3B and acidocin B represent group i and group ii circular bacteriocins, respectively. Lacticin Q and LsbB represent saposin fold exhibiting and single helical structure exhibiting single peptide leaderless bacteriocins, respectively. Enterocin 7 (Ent 7A and Ent 7B) represents the saposin fold exhibiting two-peptide leaderless bacteriocins. Red indicates hydrophobic residues and white represents hydrophilic resides. The color intensity indicates the hydrophobicity or hydrophilicity of the residues. Images are generated using PyMol Molecular Graphics System.

Unexpectedly, despite the lack of significant sequence homology and obvious disparity in their length relative to circular bacteriocins, the majority leaderless bacteriocins display a similar structural saposin fold (**Figures 5C,E**) (Lohans et al., 2013; Acedo et al., 2016). This characteristic structural motif of saposins is reportedly found in a larger superfamily of proteins known as saposin-like peptides and now including group i circular and some leaderless bacteriocins (Towle and Vederas, 2017). Apparently, this particular structural motif are surprisingly present in a wide range of heterologous and unrelated peptides. One may wonder if these peptides are genetically related in the course of its evolutionary descend. However, unlike native saposins and related peptides, which possess intramolecular disulfide linkages between cysteine residues, circular and leaderless bacteriocins do not have any disulfide bonds to stabilize their structural fold. These intermolecular bonds found in saposins is responsible in giving stability to the molecules. Whereas in the case of circular and leaderless bacteriocins, the stability of the fold is conferred by the head-to-tail covalent bonding (only true for circular bacteriocins) (Himeno et al., 2015) and the hydrophobic and electrostatic interactions of the molecules (Towle and Vederas, 2017). The distinct structural confirmation of saposins and saposin-like peptides is said to play a role in their membrane interacting biological activity (Ahn et al., 2003). Coincidentally, as discussed above, the ability of both circular and leaderless bacteriocins to interact with the bacterial membrane is crucial for their antimicrobial action. Furthermore, superimposition of the structure of the saposin fold exhibiting bacteriocins with saposin C showed remarkable similarity in the orientation of their structures despite the absence of significant similarity in their sequences and lengths (**Figure 6**). It would be interesting to experimentally verify the correlation between the structural consensus shared among these bacteriocins and their mode of action. However, it is worth noting that most circular and leaderless bacteriocins are highly cationic peptides (**Tables 1**, **2**). It has been proposed that saposin fold exhibiting bacteriocins interact with the bacterial membrane through electrostatic interaction (Towle and Vederas, 2017). It is also said that the hydrophobicity of these peptides is crucial in membrane pore formation, particularly the peptides with low overall surface charge such as group ii circular bacteriocins (**Table 1**) (Towle and Vederas, 2017). Moreover, rendering of the hydrophobicity surface plots of these bacteriocins show apparent clusters of hydrophobic patches in their surfaces. These patches are more distinctly exhibited in circular bacteriocins than leaderless bacteriocins (**Figure 7**). This data is in perfect agreement with the hydrophobicity index exhibited by these bacteriocins (**Tables 1**, **2**). Thus far, the majority of circular and leaderless bacteriocins have been shown to disrupt bacterial membranes and cause cellular component leakage through pore formation. However, there are still some disagreement on whether a receptor molecule is required for their mode of action as in the case of the circular garvicin ML. Whereas the receptor molecule-requiring-mode of action of the leaderless bacteriocin, LsbB, should not be attributed to the above-mentioned structural consensus since this bacteriocin does not display such a fold. LsbB, and its related leaderless bacteriocins (Enterocins K1, Q, and EJ97), assume a single α-helical structure at the N-terminal with an unstructured C-terminal region rather than the above-mentioned saposin-like fold (**Figure 5D**) (Ovchinnikov et al., 2014).

## Applications, Challenges, and Prospects

Bacteriocins, particularly from LAB, have been traditionally utilized in many food preservation-related technologies. However, their application areas have since then expanded to different industries. The rapidly growing concern on the emergence of multidrug resistant pathogens, coupled with the shrinking repertoire of effective antibiotics triggered the desperate search for new alternative antimicrobial agents. Due to the unique antimicrobial mechanisms of bacteriocins that enable them to not discriminate between antibiotic resistant and sensitive strains, bacteriocins have been considered as a viable alternative to conventional antibiotics (Cotter et al., 2013). Additionally, with the rapid development of advanced research tools, particularly in the field of genomics and nanotechnology, more fascinating applications of bacteriocins in the health industry have emerged, particularly their potential use as novel carrier molecules (Chikindas et al., 2018). Furthermore, other equally interesting application of bacteriocins across different industries have been described as a consequence to the reported diverse bioactivities of some bacteriocins. A few select bacteriocins showed some potential as antiviral agents, as plant growth promoters, and as anti-cancer agents (Drider et al., 2016). Recently, it was reported that enterocin AS-48 showed promising bioactivity against the protozoan parasites of the genera *Leishmania* (Abengózar et al., 2017) and *Trypanosoma* (Martínez-García et al., 2018).

However, there are still a number of unresolved challenges that hindered the large-scale utilization of bacteriocin in various industries despite their enormous potential. Understanding the biology of bacteriocins, particularly the molecular mechanistic details of their production, regulation, immunity, and mode of action, has remained the primary prerequisite for the full realization of their potential. Moreover, an economically feasible production system for its sufficient scale and quality production is necessary to propel the industrial scale utilization of bacteriocins across industries. Nonetheless, with the rapid expansion of research dedicated to bacteriocins, both in quality and quantity, novel approaches that can address these challenges will surely be developed in the near future.

In the case of circular bacteriocins, their broad-spectrum bioactivity and exceptional stability are favorable characteristics making them an ideal choice for their utilization in the above-mentioned application areas. However, the molecular mechanism of their circularization still holds the most promise for their application, as such knowledge can be utilized as scaffolds in the design of stable drugs through genetic engineering (Craik et al., 2010; Jagadish and Camarero, 2010). Recently, a chemoenzymatic approach in producing circular peptides from linear precursors has been reported. The process utilized an enzyme, termed butelase 1, a plant-derived enzyme responsible in the circularization of a cyclotide (Nguyen et al., 2014; Hemu et al., 2016). Although the strategy appears to be effective in circularizing a linear precursor, the issue on its cost effectiveness, particularly in the assembly of the linear precursor molecule in large scale set-up could undermine the practicality of the system. From an economic standpoint, the much cheaper microbial-based production systems still remain the preferred choice for large-scale production of such biomolecules. Hence, more efforts should be done for the characterization of the biosynthetic enzymes, particularly the identification of their functional domains, in order to fully understand the underlying mechanisms of circular bacteriocins biosynthesis.

On the other hand, the application of leaderless bacteriocins across different industries are equally promising. Some leaderless bacteriocins show bioactivity even at very low concentrations. Lacticin Q displays strong bioactivity even at nanomolar concentrations whereas many other non-leaderless bacteriocins are active at a relatively higher concentration (Fujita et al., 2007). Although some leaderless bacteriocins are produced by a number of strains that are often associated with pathogenicity and having structural similarity with hemolytic peptides and toxins (i.e., aureocin A53 and A70 are produced by *S. aureus*), these leaderless bacteriocins have been demonstrated to have no cytotoxic and hemolytic activities, thus underlining the safety of their application (Fagundes et al., 2016a,b). Similar observation was manifested by enterocin DD14 (identical to enterocins 7 and MR10) when it was evaluated for its cytotoxicity against intestinal epithelial cell line IPEC-1 (Caly et al., 2017). But perhaps the “simplicity” of the structure of leaderless bacteriocins holds their enormous industrial potential. Since they do not possess any leader sequence and do not undergo any posttranslational processing, leaderless bacteriocins can be easily introduced to existing recombinant expression systems either or both prokaryotic and eukaryotic hosts by just the mere cloning of their structure genes. They can also be expressed using the general secretion pathways of their host by simply fusing the host-specific N-terminal signal peptide into the bacteriocin thus enabling the host to secrete the active bacteriocin through its *Sec*-pathway. As already mentioned above, enterocin L50A and L50B have been successfully produced using the yeast *Saccharomyces cerevisiae* and *Pichia pastoris* through the general secretion systems of these eukaryotic hosts. The yeast mating pheromone α-factor 1, MFα1, directed the efficient secretion of the bacteriocins through the *Sec*-system of both hosts cells when it was fused to the N-terminal region of the bacteriocins (Basanta et al., 2009, 2010). In both cases, the successful expression of the bacteriocins in these eukaryotic hosts were done without the expression of the bacteriocin’s cognate immunity proteins as most bacteriocins require much higher concentration to exert bioactivities against eukaryotic cells.

## Conclusion

Bacteriocins, particularly from LAB, are excellent antimicrobials that are traditionally appreciated for their effectiveness in food preservation. However, in the past decade, an extra attention geared toward their possible application in the health industry was apparent. Their viability as an alternative to conventional antibiotics, particularly for the control of multi-drug resistant pathogens is now being evaluated. A growing number of bacteriocins belonging to the group known as circular and leaderless bacteriocins possessing unique features both in their structures and biosynthetic mechanisms are increasingly attracting more attention due to their enormous industrial potential. However, there is still so much to know about their biosynthesis and mode of action in order to materialize their full potential. A better understanding on how these antimicrobials inhibit their target bacteria is important in minimizing possible development of resistance mechanisms in the future. Furthermore, knowledge on the mechanisms of their self-immunity could serve as a reference point in their bioengineering to further curtail any future resistance mechanism among their targets. But with the rapid development in science and technology, particularly in fields of genomics, genetic engineering, structure biology and biotechnology, a better understanding on the biology of their origin and molecular nature should be attained sooner and this would facilitate for their large-scale application across industries.

## Author Contributions

All authors defined the topic of the review. RP drafted the manuscript. TZ and KS polished it. All authors read and approved the final manuscript.

## Conflict of Interest Statement

The authors declare that the research was conducted in the absence of any commercial or financial relationships that could be construed as a potential conflict of interest.
